# Optimized microwave illusion device

**DOI:** 10.1038/s41598-017-04410-4

**Published:** 2017-06-21

**Authors:** Benjamin Vial, Max Munoz Torrico, Yang Hao

**Affiliations:** School of Electronic Engineering and Computer Science, Queen Mary University of London, London, E1 4NS United Kingdom

## Abstract

We report the design, fabrication and experimental verification of an illusion device working at microwave frequencies. A two dimensional topology optimization procedure is employed to find the binary layout of a dielectric coating that, when wrapped around a metallic cylinder, mimics the scattering from a predefined, arbitrarily-shaped dielectric object. Fabrication is carried out with 3D-printing and spatially resolved near field measurements in a waveguide configuration were performed, allowing us to map the illusion effect. Our work provides general guidelines for engineering electromagnetic illusions but can be extended to shape the near and far-field radiations using low index isotropic materials.

## Introduction

During the past ten years, considerable attention has been devoted to the study and application of Transformation Optics (TO)^1, 2^. Based on geometric transformations, the idea provide a powerful theoretical framework to manipulate *ad libitum* the propagation of electromagnetic waves. Generally, the applied change of coordinates amounts to replacing the initial materials by equivalent inhomogeneous anisotropic materials^3^. From a practical point of view, those exotic properties can be mimicked by metamaterials with suitably designed unit cells^4^. However, most of the TO-based media require conducting or plasmonic inclusions, but the propagation loss represent a major limitation for practical applications, particularly at visible wavelengths.

Certainly the most studied application of TO rely on the idea that an object surrounded by a coating consisting of a material with specially tailored properties becomes invisible to electromagnetic waves^5–18^. The device that implements this phenomenon has been called an “invisibility cloak”, in reference to Harry Potter, the popular character of J. K. Rowling. But the young wizard has other spells beside his famous cloak. One of the most impressive is the “polyjuice potion” that is able to turn somebody into anybody else’s appearance. This concept as been translated and verified theoretically and experimentally by applying TO to create illusions^19–23^, i.e. to design a device that gives an arbitrary predefined electromagnetic response to any object placed inside it.

However, as mentioned before, the complexity and limitations of the material properties derived from TO have called for other approaches for practical applications. One route employed to engineering specific wave behaviour is topology optimization^24, 25^. It has been successfully applied to design devices such as invisibility cloaks^26–33^, photonic crystals based devices^34–36^, metamaterials^37–40^, frequency selective surfaces^41^ or antennas^42–45^, using various strategies such as local gradient-based approaches or global stochastic algorithms.

Recent advances in additive manufacturing, whereby a component is built up layer by layer, open up the design domain significantly and relax fabrication constraints. Techniques such as 3D-printing allows to realize components with intricate complexities, enabling the production of optimal microwave materials and devices with improved performances^41, 46–49^.

Herein we report the numerical simulation, optimization, manufacturing and experimental validation of an illusion device at microwave frequencies. The key idea is that we want to make a metallic cylinder (or any object placed inside a perfectly conducting shell with the same outer diameter) behave like a predefined, arbitrary shaped dielectric object (the reference) for a given excitation. A 2D finite element model is employed to simulate the electromagnetic response of the considered structures excited by a Transverse Electric (TE) polarized line source. We use the polymer acrylonitrile butadiene styrene (ABS), a standard thermoplastic used in commercial 3D printers as our low index dielectric material. A gradient based topology optimization algorithm is used to find the layout of a dielectric shell that minimizes the difference between the fields scattered by the illusion and reference structures. The obtained design and reference object were then 3D-printed by a stereolithography based process. The electric field maps of the various configurations are measured in a waveguide configuration and show a good agreement compared too simulated results, demonstrating the illusion effect. We believe this work may serve for designing improved microwave devices such as antennas or sensors where one can control the near and far fields in a predefined manner, while incorporating fabrication and material constraints necessary for real life applications.

## Results

### Optimized illusion device

**Figure 1:**
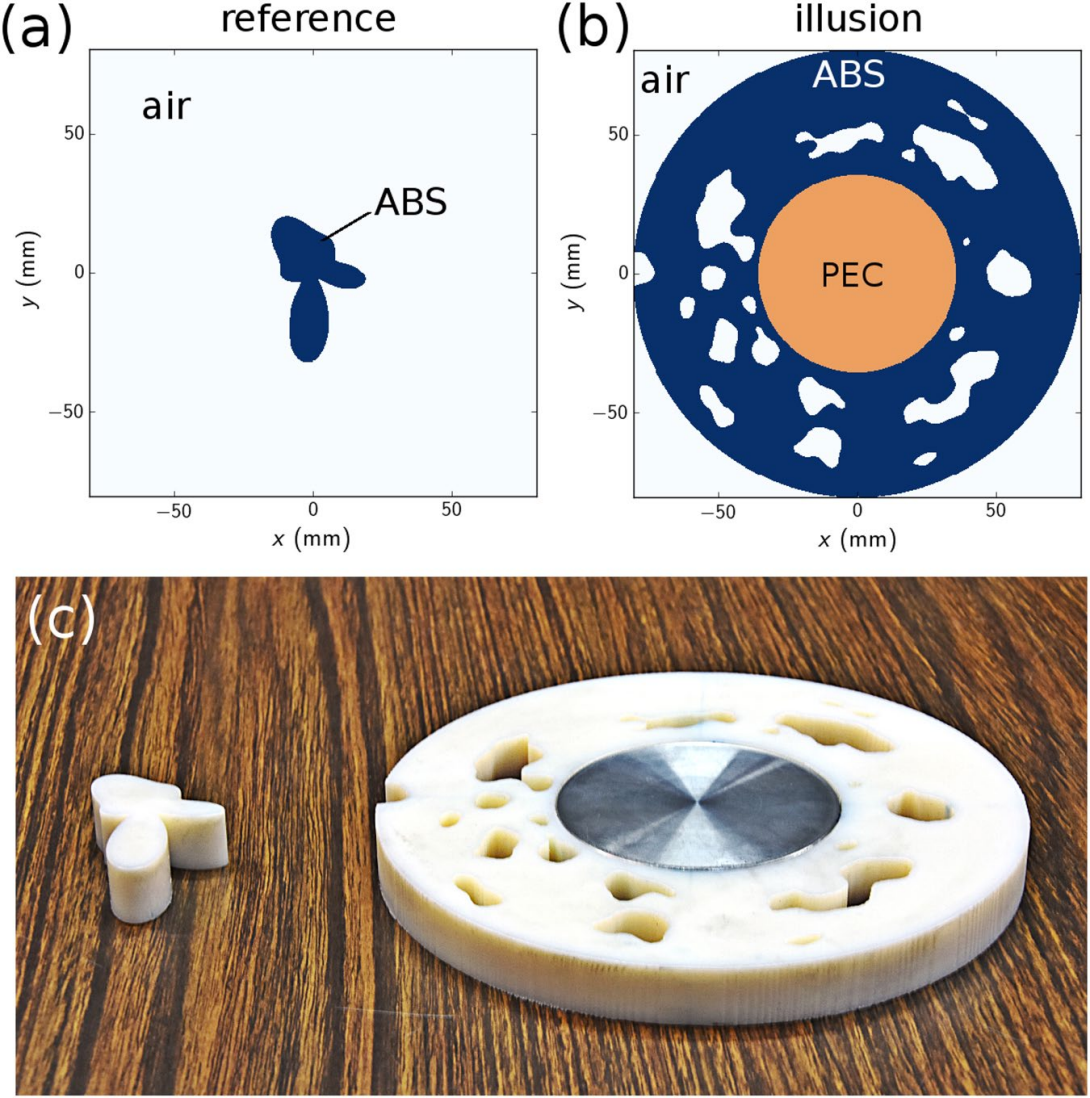
Optimization results. (**a**) Reference dielectric object. (**b**) Optimized illusion device. (**c**) Photograph of the 3D printed structures.

As to prove the versatility of the design process, our reference object has an arbitrary complex shape described by Equation(3) and is depicted on Fig. 1(a). It is made of ABS, the same material that we use for the illusion cover, although this is not mandatory nor a constraint of the method. We run the optimization process for various uniform initial densities (see Methods) and obtain different designs. Because of the nature of our optimization algorithm, starting with different material distribution yields different solutions as the problem exhibits many local minima. We retain the design illustrated on Fig. 1(a) as it is a single piece contiguous domain for obvious practical reasons. The resulting 2D objects are extruded by 14 mm in the *z* direction and then 3D printed (see Methods). A photograph of the samples is shown on Fig. 1(c), proving the ability to built complex structures using additive manufacturing techniques.

### Near field

**Figure 2:**
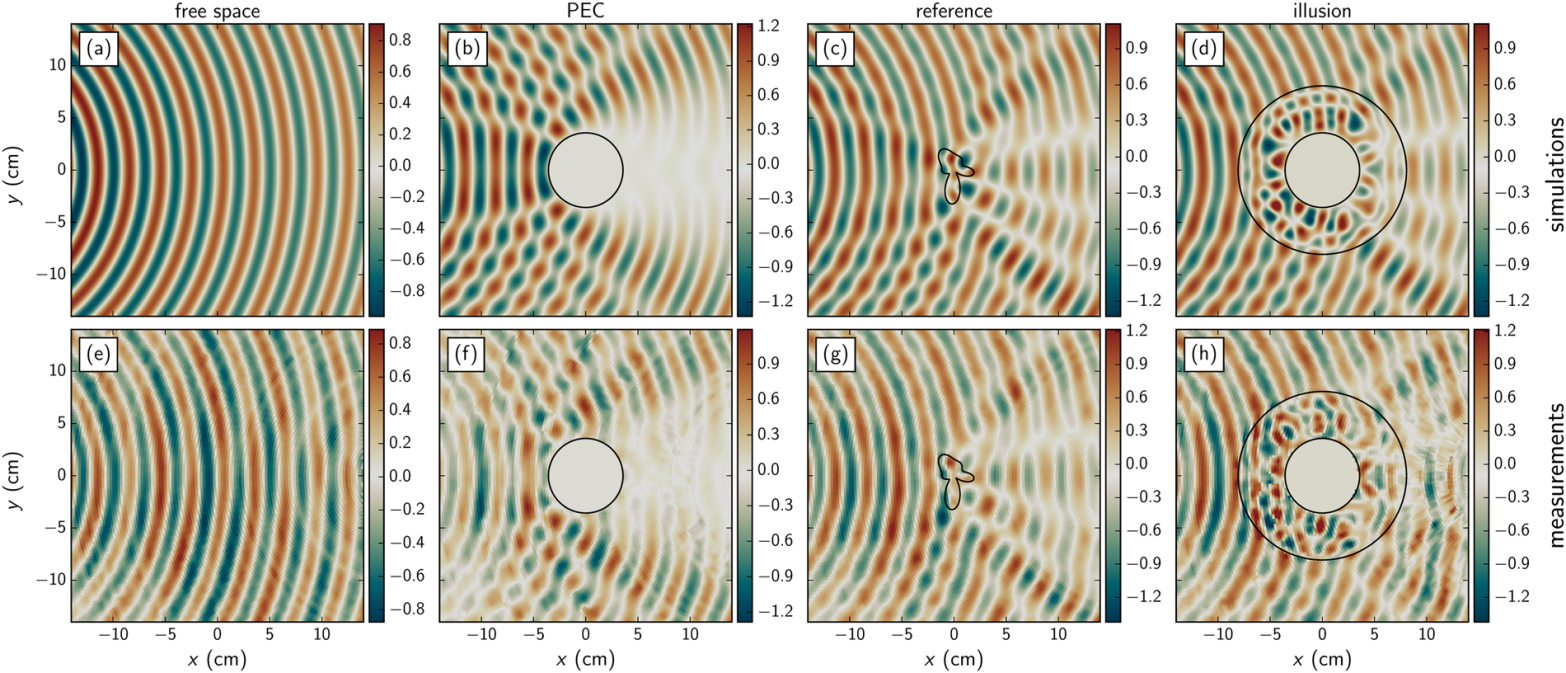
Field maps. Real part of the dielectric field at the design frequency *f* = 10 GHz. Top row (**a**–**d**) FEM simulations. Bottom row (**e**–**h**) measurements. Various case are considered: free space (**a**,**e**), PEC cylinder (**b**,**f**), reference dielectric object (**c**,**g**) and the same PEC cylinder with the proposed illusion device (**d**,**h**).

As described in Methods, we map experimentally the electric field in a waveguide to illustrate the illusion effect. Our measurement results are reported on Fig. 2(e–h) and compared with simulations (a–d). We first consider the empty waveguide and obtain a cylindrical propagating wave with excellent agreement with simulations (the free space 2D Green’s function). This indicates that the line source we used in simulations is a fairly good approximation of the waveguide horn of our experiments. We then map the field in the case of the PEC cylinder, which shows a strong scattering and a shadow region in the forward direction as expected from theory. Due to its asymmetrical shape, the reference dielectric object diffracts the incident wave in a characteristic pattern. The measured values agree really well with simulations results and prove our ability to map complex diffracted fields with sub-wavelength resolution. Finally, the illusion device wrapped around the metallic cylinder shows a scattering in free space very similar to the reference object. The measured electric field matches the reference field as expected from the numerical optimization, more particularly the shadow caused by the PEC cylinder is reduced and the characteristic asymmetric pattern is retrieved.

**Figure 3:**
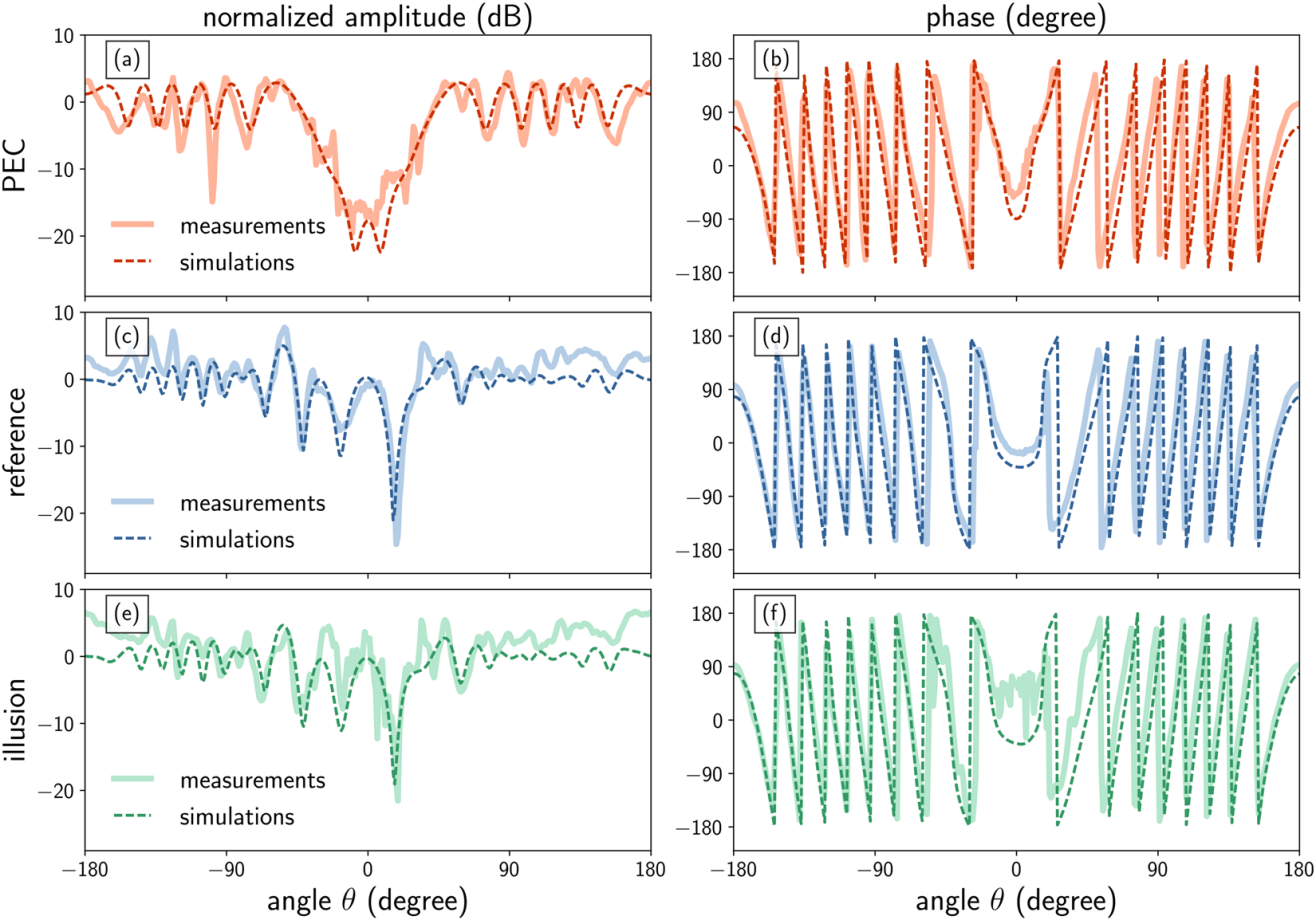
Amplitude and phase. Electric field magnitude normalized to the free space value (in dB, left column) and phase (in degree, right column) for the PEC cylinder (**a**,**b**), the reference dielectric object (**c**,**d**) and the illusion device (**e**,**f**). The measured (thick solid lines) and computed (thin dashed lines) values are plotted as a function of the observation angle *θ* (in degree) for a fixed radius *r* = 1.5 *R*_ill_.

To illustrate further these results, we plot cuts of those field maps on Fig. 3 at a constant radius *r* = 1.5*R*_ill_. The amplitude normalized to the incident field and phase are displayed for measurements (thick solid lines) and simulations (thin dashed lines) and are in fairly good agreement. As mentioned before, the PEC cylinder provoke a strong attenuation in the forward direction (*θ* = 0 degree), and backscattering. As for the reference and illusion cases, their amplitude patterns show strong similarities, particularly in the forward direction where we can observe a symmetric angular response in both cases. Reference and illusion phase patterns are in good agreement and mostly differ from the PEC case in the forward direction, albeit some noise in the measured data for the illusion case.

### Performances

**Figure 4:**
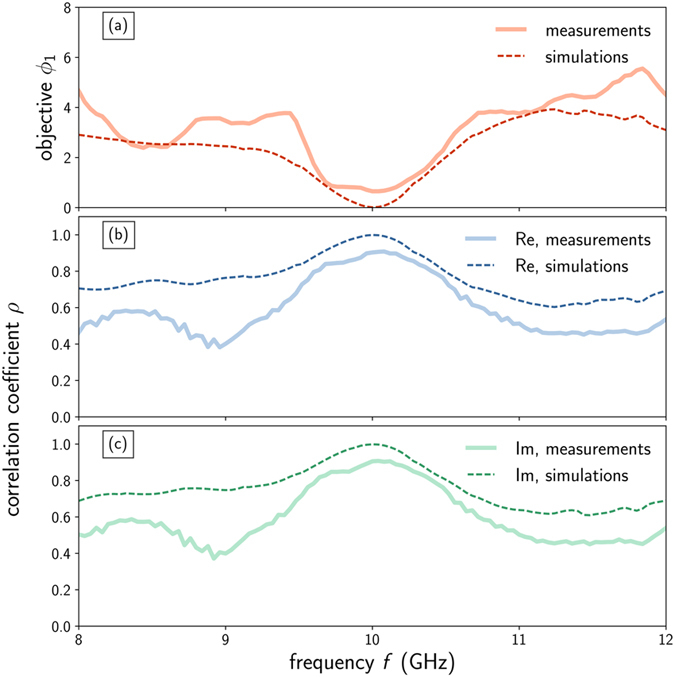
Performances of the illusion device. Measured (thick solid lines) and computed (thin dashed lines) figures of merit as a function of frequency *f*: partial objective function *ϕ*_1_ representing the relative error between the reference and illusion electric fields (**a**), and correlation coefficient between those fields for the real (**b**) and imaginary (**c**) parts.

In order to confirm the previous qualitative observations, we calculate two figures of merit to asses the performances of the illusion device. The first one is the partial objective function *ϕ*_1_ (defined in Equation 5) which is essentially the relative error between the electric fields for the reference and illusion cases. As can be seen on Fig. 4(a), *ϕ*_1_ exhibits a dip in frequency at 10 GHz, showing that the difference is minimal between the reference and illusion fields. While the simulated value is 0.01, the measured one is around 0.6, which is mainly due to experimental errors and to the local nature of this figure of merit.

Another parameter suitable to quantify the performance of the illusion device is the correlation coefficient^50^ defined as:

1 $$\rho (X,Y)=\frac{{\rm{E}}(XY)-{\rm{E}}(X){\rm{E}}(Y)}{\sqrt{{\rm{E}}({X}^{2})-{\rm{E}}{(X)}^{2}}\sqrt{{\rm{E}}({Y}^{2})-{\rm{E}}{(Y)}^{2}}}$$

where *X* = *E*_ref_ is the field produced by the reference dielectric object and *Y* = *E*_ill_ is the field scattered by the PEC cylinder covered by the illusion device. We calculated this coefficient for both real and imaginary parts of the fields and plot it as a function of frequency on Fig. 4(b) and (c) respectively. In both cases, it reaches its maximum at the design frequency of 10 GHz, with an experimental value around 0.9, while the theoretical simulated value is 0.99 (a coefficient of 1 means that both fields are perfectly correlated). This again prove that our device reproduces the desired scattering with good fidelity.

## Discussion

We designed an illusion device made of a relatively thin (1.5*λ*) low index dielectric cover with an optimized topology. When wrapped around a given PEC cylinder its function is to emulate the scattering of a predefined, arbitrarily shaped dielectric object so that an observer probing the electromagnetic field outside of the device is tricked into thinking that he detects the said reference object. Note that if the PEC cylinder is replaced by a thin PEC surface, the device will produce the illusion for *any object places inside* as the field does not penetrate. This device has been fabricated using 3D printing using a standard stereolithography process and common ABS thermoplastic material. The illusion effect is present in the near field as evidences experimental by spatially resolved waveguide measurements. The performances of the device have been assessed by two figures of merit and are in reasonable accordance with the numerical solutions. Although the illusion effect has been proven here experimentally at microwave frequencies, the design methodology is general and suitable for the optical domain. Furthermore, it only requires a low index dielectric and doe not necessitate anisotropic or plasmonic materials. We believe that the optimization route used here might be of interest for the design of other devices and materials with enhanced characteristics able to control the propagation of electromagnetic waves, not only in the far field but in the near field. The development and refinement of additive manufacturing process, including the ability to use different materials, allows the design of topologies difficult to realize with traditional fabrication techniques and claim for innovative engineering approaches. Advanced numerical techniques, efficient global optimization algorithms and innovative strategies need to be developed in order to improve the design process and the designed structures, by including multiple objectives, constraints, multiple materials and physics. It is believed that this could benefits applications such as telecommunications, antennas, sensors and energy harvesting to name a few.

## Methods

### Numerical simulations: electromagnetic problem and optimization procedure

The problem at stake is governed by Maxwell’s equation in time-harmonic regime (with convention ext (−*iωt*) discarded hereafter). The material parameters do not depend on *z* and this medium is excited by a line source located at **r**′ = (*x*′, *y*′)^T^ and directed along the *z* axis. This is the so called TE propagation with the total electric field **E** = *E* **z** satisfying the following scalar wave equation:

2 $$\nabla \cdot ({\mu }^{-1}\nabla E)+{k}^{2}\varepsilon E=\delta ({\bf{r}}{\boldsymbol{^{\prime} }}),$$

where *ε* and *μ* are the relative permittivity and permeability respectively, *δ* is the Dirac delta distribution, **r** = (*x*, *y*)^T^ is the spatial position vector and *k* = *ω*/*c* is the free space wavenumber.

**Figure 5:**
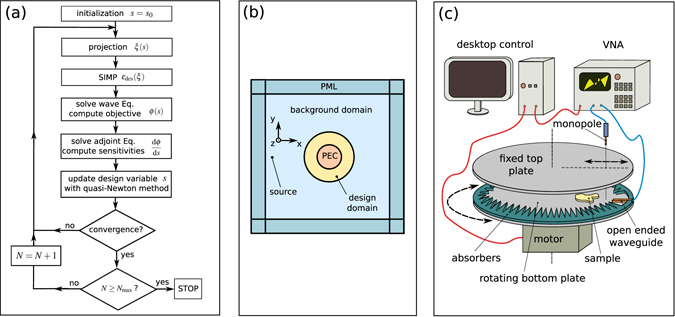
Numerical and experimental methods. (**a**) Optimization algorithm workflow. (**b**) Computational setup for the FEM optimization problem. (**c**) Measurement setup.

To solve this problem numerically, we use a Finite Element Method^51^ (FEM) commercial package (COMSOL Multiphysics). A schematics of the problem under study is given in Fig. 5(b). The line source is located at *x*′ = −260 mm and *y*′ = 0 mm. The background domain is truncated by cartesian Perfectly Matched Layers^52, 53^ (PML) in order to damp propagating waves, with thickness *t*_pml_ = *λ*_0_, constant stretching coefficients along *x* and *y* (*s*_*x*_ = *s*_*y*_ = 1 − *i*), and homogeneous Neumann condition on their outward boundaries.

For this work, we used ABS thermoplastic commonly used for 3D printing as our dielectric material, with a permittivity *ε*_ABS_ = 2.6980 − 0.0068*i* as measured at the operating frequency *f*_0_ = 10 GHz (see Methods). Furthermore, all materials are considered non magnetic (*μ* = 1).

The aim of the proposed illusion device is to design a dielectric cover that will make a metallic cylinder of radius *R*_cyl_ = 35.7 mm look like an arbitrarily shaped dielectric. The shape of this reference object is given in polar coordinates (*R*, *θ*) as a Fourier series:

3 $$R(\theta )={R}_{0}+\sum _{n\mathrm{=1}}^{5}{A}_{n}\,\cos (n\theta )+{B}_{n}\,\sin (n\theta ),$$

with *R*_0_ = 15 mm, *A* = (−0.83, −4.50, 4.02, 4.44, −0.09) mm and *B* = (−0.11, −1.62, 4.00, −1.31, −3.89) mm.

The objective is to minimize the difference between the field *E*_ill_ scattered by the PEC cylinder covered by the illusion layer and the field *E*_ref_ produced by the reference object in the background domain **Ω**_b_. Defining the integral quantities

4 $$I={\int }_{{{\rm{\Omega }}}_{{\rm{b}}}}{|{E}_{{\rm{ill}}}-{E}_{{\rm{ref}}}|}^{2}{\rm{d}}{\bf{r}},{\rm{and}}\quad {I}_{{\rm{ref}}}={\int }_{{{\rm{\Omega }}}_{{\rm{b}}}}{|{E}_{{\rm{ref}}}|}^{2}{\rm{d}}{\bf{r}},$$

the objective function to minimize is

5 $${\varphi }_{1}({\varepsilon }_{{\rm{des}}})=I/{I}_{{\rm{ref}}}$$

where *ε*_des_ is the permittivity in the design domain (an annular layer of thickness *t*_des_ = 1.5 *λ*_0_ = 45 mm), subject to constraints *ε*_min_ ≤ *ε*_des_ ≤ *ε*_max_, with *ε*_min_ = *ε*_air_ = 1 and *ε*_max_ = *ε*_ABS_ = 2.6980 − 0.0068*i*.

In addition, we set additional constraint on the gradient of permittivity to avoid chessboard patterns and very small features by defining a second objective function

6 $${\varphi }_{2}({\varepsilon }_{{\rm{des}}})=\frac{{h}_{{\rm{mesh}}}^{2}}{S}{|\nabla {\varepsilon }_{{\rm{des}}}|}^{2},$$

where *S* = *πt*_des_ (*t*_des_ + 2*R*_cyl_) is the area of the design domain and *h*_mesh_ is the maximum size of a mesh element.

Following the so-called solid isotropic material interpolation (SIMP)^54^, we define a density function *ξ* such that

$${\varepsilon }_{{\rm{des}}}(\xi )={\varepsilon }_{{\rm{\min }}}+{\xi }^{p}({\varepsilon }_{{\rm{\max }}}-{\varepsilon }_{{\rm{\min }}})$$

where *p* is a penalty factor (*p* = 1 here).

In order to enforce a binary design, we apply a threshold projection^55^ at each global iteration:

$$\xi (s)=\frac{\tan \,{\rm{h}}(\beta \nu )+\,\tan \,{\rm{h}}(\beta (s-\nu ))}{\tan \,{\rm{h}}(\beta \nu )+\,\tan \,{\rm{h}}(\beta \mathrm{(1}-\nu ))},$$

with *ν* = 1/2 and *β* = 2^*N*^ where *N* < *N*_max_ is the number of optimization global iteration (we set *N*_max_ = 8).

The topology optimization problem is thus to minimize the functional *ϕ* of the final design variable *s*

7 $$\varphi (s)=\gamma \,{\varphi }_{1}(s)+\mathrm{(1}-\gamma )\,{\varphi }_{2}(s),$$

subject to constraints 0 ≤ *s* ≤ 1 and where *γ* is a weight parameter between 0 and 1 (*γ* = 0.75 here). The gradient-based optimization code uses the Quasi-Newton method^56^ to update the design iteratively, starting from an initial homogeneous density *s*_0_ = 0.7. The sensitivity of the objective function with respect to *s* is calculated by the adjoint method^36^. The problem is discretized by the FEM using second order Lagrange elements and the solutions are computed with a direct solver^57^. An overview of the optimization algorithm is given on Fig. 5(a).

### Fabrication and Material Characterisation

The samples were 3D printed using an Objet30 Prime from Stratasys. The printer uses a polyjet technique to build smooth and accurate 3D models. The building process combines the techniques of inkjet and stereolythography (SLA) printing methods. The tiny droplets of liquid photopolymer are cured with a UV laser beam which instantly solidify the photopolymer creating the layers of the building model. Each of these layers has an accuracy down to 100 microns. For this particular experiment, VeroWhitePlus (RGD835) material was used. Three different models of 60 × 60 × 3 mm were manufactured using a high-resolution and a glossy finish. The printing environment was configured so the support material is built around the sample. Once the printing is finished, the gel-like support is easily removed using a high pressure water flow. The prototypes were left to dry for a couple of days thus water effects do not cause any type of uncertainties on the material characterisation.

The dielectric properties were measured using an 85072A Split-Cylinder from Keysight Technologies. The instrument operates at 10 GHz with a high Q, producing good loss tangent resolution thanks to the adjustable electrical coupling into the cavity. The latter resonator is connected to a 5230C PNA-L Network Analyser, from the same supplier, which runs the 85071E Materials Measurement Software. Multiple measurements were taken for each sample with an IF bandwidth of 100 Hz. The average results from 5 independent measurements yield a 2.698 for the real part of the complex permittivity and 0.0068 for the imaginary part.

### Experimental setup

A near-field scanning system operating at frequencies between 6 and 12 GHz is used to map the electric field (see Fig. 5(c)). It is composed of two parallel conducting plates, each with a diameter of 1 m and spaced 15 mm apart. This ensures that the only propagating mode is the fundamental TEM mode for frequencies bellow 10 GHz. The source is an X-band waveguide (single-mode operational frequency range: 8.2–12.4 GHz; cutoff frequency: 6.557 GHz) fixed to the rotating bottom plate. It produces a cylindrical wave from its end, similar to a line source excitation. Microwave absorbers are added at the boundaries of the system in order to reduce scattering and reflections from the edges. Holes have been drilled along the radius of the fixed top plate with a spatial resolution of 5 mm. A monopole probe connected to the first port of a vector network analyzer (VNA) measures the amplitude and phase of the S-parameters with respect to the source waveguide connected to the other port. By varying the position of the probe along the radius and rotating the bottom plate, we obtain a 2D polar map of the near field. A computer controls and synchronizes the motor and the VNA through a LabView code.

## References

[1] Pendry JB, Schurig D, Smith DR (2006). Controlling electromagnetic fields. Science.

[2] Leonhardt U (2006). Optical conformal mapping. Science.

[3] Zolla F, Guenneau S, Nicolet A, Pendry JB (2007). Electromagnetic analysis of cylindrical invisibility cloaks and the mirage effect. Opt. Lett..

[4] Chen H, Chan CT, Sheng P (2010). Transformation optics and metamaterials. Nat. Mater..

[5] Cai W, Chettiar UK, Kildishev AV, Shalaev VM (2007). Optical cloaking with metamaterials. Nat. Photonics.

[6] Li C, Li F (2008). Two-dimensional electromagnetic cloaks with arbitrary geometries. Opt. Express.

[7] Schurig D (2006). Metamaterial electromagnetic cloak at microwave frequencies. Science.

[8] Dupont G (2009). Revolution analysis of three-dimensional arbitrary cloaks. Opt. Express.

[9] Nicolet A, Zolla F, Guenneau S (2008). Electromagnetic analysis of cylindrical cloaks of an arbitrary cross section. Opt. Lett..

[10] Ni X, Wong ZJ, Mrejen M, Wang Y, Zhang X (2015). An ultrathin invisibility skin cloak for visible light. Science.

[11] Gharghi M (2011). A carpet cloak for visible light. Nano Lett..

[12] Fischer J, Ergin T, Wegener M (2011). Three-dimensional polarization-independent visible-frequency carpet invisibility cloak. Opt. Lett..

[13] Valentine J, Li J, Zentgraf T, Bartal G, Zhang X (2009). An optical cloak made of dielectrics. Nat. Mater..

[14] Ergin T, Fischer J, Wegener M (2011). Optical phase cloaking of 700 nm light waves in the far field by a three-dimensional carpet cloak. Phys. Rev. Lett..

[15] Li J, Pendry JB (2008). Hiding under the carpet: A new strategy for cloaking. Phys. Rev. Lett..

[16] Ergin T, Stenger N, Brenner P, Pendry JB, Wegener M (2010). Three-dimensional invisibility cloak at optical wavelengths. Science.

[17] Leonhardt U, Tyc T (2009). Broadband invisibility by non-euclidean cloaking. Science.

[18] Liu R (2009). Broadband ground-plane cloak. Science.

[19] Nicolet, A., Zolla, F. & Geuzaine, C. Generalized Cloaking and Optical Polyjuice. *Proceedings of the 8th conference on Electrical, Transport and Optical Properties of Inhomogeneous Media* (2009).

[20] Jiang WX, Ma HF, Cheng Q, Cui TJ (2010). Illusion media: Generating virtual objects using realizable metamaterials. Appl. Phys. Lett..

[21] Jiang WX, Cui TJ (2011). Radar illusion via metamaterials. Phys. Rev. E.

[22] Lai Y (2009). Illusion optics: The optical transformation of an object into another object. Phys. Rev. Lett..

[23] Li C (2010). Experimental realization of a circuit-based broadband illusion-optics analogue. Phys. Rev. Lett..

[24] Sigmund O, Maute K (2013). Topology optimization approaches. Struct. Multidiscip. Optim..

[25] Jensen JS, Sigmund O (2011). Topology optimization for nano-photonics. Laser Photon. Rev.

[26] Andkjær J, Sigmund O (2011). Topology optimized low-contrast all-dielectric optical cloak. Appl. Phys. Lett..

[27] Andkjær J, Asger Mortensen N, Sigmund O (2012). Towards all-dielectric, polarization-independent optical cloaks. Appl. Phys. Lett..

[28] Urzhumov Y, Landy N, Driscoll T, Basov D, Smith DR (2013). Thin low-loss dielectric coatings for free-space cloaking. Opt. Lett..

[29] Yamada T, Watanabe H, Fujii G, Matsumoto T (2013). Topology optimization for a dielectric optical cloak based on an exact level set approach. IEEE Trans. Magn..

[30] Otomori M (2013). Level set-based topology optimization for the design of an electromagnetic cloak with ferrite material. IEEE Trans. Magn..

[31] Fujii G, Watanabe H, Yamada T, Ueta T, Mizuno M (2013). Level set based topology optimization for optical cloaks. Appl. Phys. Lett..

[32] Lan L, Sun F, Liu Y, Ong C, Ma Y (2013). Experimentally demonstrated a unidirectional electromagnetic cloak designed by topology optimization. Appl. Phys. Lett..

[33] Vial B, Hao Y (2015). Topology optimized all-dielectric cloak: design, performances and modal picture of the invisibility effect. Opt. Express.

[34] Jensen JS, Sigmund O (2004). Systematic design of photonic crystal structures using topology optimization: Low-loss waveguide bends. Appl. Phys. Lett..

[35] Sigmund O, Jensen JS (2003). Systematic design of phononic band–gap materials and structures by topology optimization. Philos. Trans. A. Math. Phys. Eng. Sci..

[36] Jensen JS, Sigmund O (2005). Topology optimization of photonic crystal structures: a high-bandwidth low-loss t-junction waveguide. J. Opt. Soc. Am. B.

[37] Zhou S, Li W, Chen Y, Sun G, Li Q (2011). Topology optimization for negative permeability metamaterials using level-set algorithm. Acta Mater..

[38] Diaz AR, Sigmund O (2009). A topology optimization method for design of negative permeability metamaterials. Struct. Multidiscip. Optim..

[39] Wang Y, Luo Z, Zhang N, Kang Z (2014). Topological shape optimization of microstructural metamaterials using a level set method. Comput. Mater. Sci..

[40] Kildishev AV (2007). Stochastic optimization of low-loss optical negative-index metamaterial. J. Opt. Soc. Am. B.

[41] Barton JH, Garcia CR, Berry EA, Salas R, Rumpf RC (2015). 3-d printed all-dielectric frequency selective surface with large bandwidth and field of view. IEEE Trans. Antennas Propag..

[42] Koziel S, Bekasiewicz A, Couckuyt I, Dhaene T (2014). Efficient multi-objective simulation-driven antenna design using co-kriging. IEEE Trans. Antennas Propag..

[43] Ourir A, de Lustrac A, Lourtioz J-M (2006). Optimization of metamaterial based subwavelength cavities for ultracompact directive antennas. Microwave Opt. Technol. Lett..

[44] Ouedraogo RO (2010). *In situ* optimization of metamaterial-inspired loop antennas. IEEE Antennas Wirel. Propag. Lett..

[45] Rahmat-Samii, Y. & Michielssen, E. (eds) *Electromagnetic Optimization by Genetic Algorithms*, 1st edn (John Wiley & Sons, Inc., 1999).

[46] Castles F (2016). Microwave dielectric characterisation of 3d-printed BaTiO3/ABS polymer composites. Sci. Rep..

[47] Deffenbaugh PI, Rumpf RC, Church KH (2013). Broadband microwave frequency characterization of 3-d printed materials. IEEE Trans. Compon. Packag. Manuf. Technol..

[48] Garcia CR (2012). 3d printing of anisotropic metamaterials. Prog. Electromagn. Res. Lett..

[49] Rumpf RC, Pazos J, Garcia CR, Ochoa L, Wicker R (2013). 3d printed lattices with spatially variant self-collimation. Prog. Electromagn. Res.

[50] Bao D, Mitchell-Thomas R, Rajab K, Hao Y (2013). Quantitative study of two experimental demonstrations of a carpet cloak. IEEE Antennas Wireless Propag. Lett..

[51] Jin, J. *The finite element method in electromagnetics* (John Wiley & Sons, 2014).

[52] Berenger J-P (1994). A perfectly matched layer for the absorption of electromagnetic waves. J. Comput. Phys..

[53] Lassas M, Liukkonen J, Somersalo E (2001). Complex Riemannian metric and absorbing boundary conditions. J. Math. Pure. Appl..

[54] BendsÃ¸e MP, Sigmund O (1999). Material interpolation schemes in topology optimization. Arch. Appl. Mech..

[55] Wang F, Lazarov B, Sigmund O (2011). On projection methods, convergence and robust formulations in topology optimization. Struct. Multidiscip. Optim..

[56] Tapia R (1977). Diagonalized multiplier methods and quasi-newton methods for constrained optimization. J. Optim. Theory. Appl.

[57] Schenk O, Wächter A, Hagemann M (2007). Matching-based preprocessing algorithms to the solution of saddle-point problems in large-scale nonconvex interior-point optimization. Comput. Optim. Appl..

